# Rare and Atypical Case of Turner Syndrome With Three Cell Lines

**DOI:** 10.7759/cureus.41128

**Published:** 2023-06-29

**Authors:** Amal Essouabni, Mohamed Ahakoud, Hayat Aynaou, Laila Bouguenouch, Houda Salhi, Ouldim Karim, Hanan Elouahabi

**Affiliations:** 1 Department of Endocrinology, Diabetology, Metabolic Diseases and Nutrition, Hassan II University Hospital, Fez, MAR; 2 Medical Genetics and Onco-genetics Laboratory, Hassan II University Hospital, Fez, MAR

**Keywords:** karyotype, dysmorphic syndrome, amenorrhea, mosaicism, turner syndrome

## Abstract

Turner's syndrome is a rare complex genetic disease characterized by gonadal dysgenesis and sexual chromosomal abnormalities. Half of the patients affected are monosomic, for the X chromosome, and for the remaining patients, a variety of chromosomal abnormalities have been reported. Only a small percentage (3%-4%) of people with Turner syndrome have triple X cell line mosaicism (47, XXX). It has been reported that patients 45, X/47, XXX have normal intelligence, a higher rate of spontaneous menstruation, an increased number of pregnancies, and a lower frequency of short stature (60%) compared to patients 45, X. In this work, we will present a rare and atypical case of a patient who presents a rare chromosomal mosaicism, with three chromosomal lineages, contrasting with a typical clinical picture of Turner syndrome.

## Introduction

Turner syndrome is a gonadal dysgenesis defined as the total or partial loss of a sex chromosome [1]. The anomalies of Turner syndrome include short stature (95%-100%), primary amenorrhea (85%), infertility (98%), and characteristic stigmata [2]. The variable expressivity of size and other physical characteristics may be only partially related to the chromosomal formula.

Approximately 50%-60% of patients have complete monosomy X (45, X). A multiplicity of chromosomal aberrations has been described for others [3]. The most frequent is the presence of an isochromosome of the long arm of X (i(Xq)) and the X-ring and mosaicism for two or more normal or abnormal cell lines (e.g., 45, X/46, XX; 45, X/46, X, i(Xq); 45, X/46, XY). A small proportion (3%-4%) [4,5] of individuals with Turner syndrome are mosaics for a triple X cell line (47, XXX).

Although most women (47, XXX) have normal ovarian function and fertility, some have delayed menarche or premature ovarian failure, and oligomenorrhea [4]. We describe a very special case of a patient, with a 47, XXX/45, X/46, XX mosaicism, who presents a phenotype and symptomatology typical of Turner syndrome.

## Case presentation

We describe the case of a patient, aged 17 years and three months, admitted in our training, for exploration and management of a stature-weight growth and pubertal delay. She was born in a non-consanguineous marriage and had a full-term pregnancy with normal progress and good psychomotor development. The parents and brothers seemed to be in good health and all had a normal height and weight. Moreover, the patient had no notable pathological history (mental retardation, chronic pathologies, or others) or similar cases in the family.

On clinical examination, her height was 143 cm (-4SD) and weight was 31 kg (-3SD), with the presence of a dysmorphic syndrome, made of a triangular face, and pectus excavatum, with nipple spacing. However, her external genitalia appeared normal without a palpable inguinal mass. Her Tanner stage was breast I, pubic hair IV, and axillary hair sparse.

**Figure 1:**
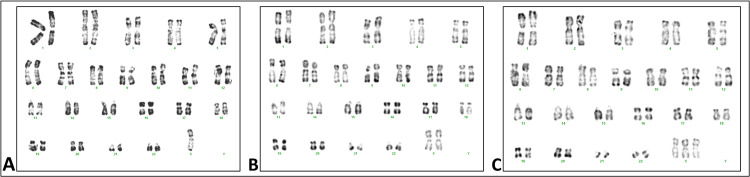
Karyotype image of our patient showing the three cell lines (A) 45, X. (B) 46, XX. (C) 47, XXX.

Faced with this clinical picture, a primary workup was requested, which came back in favor of a hypergonadotropic hypogonadism, with a bone age estimated between 12 and 13 years, according to the GREULISH and PYLE method (56-year gap from the chronological age). We completed a karyotype coming back in favor of a mosaic Turner syndrome with three cell lines: 45X/46, XX/47, XXX with negative SRY (presence of three cell populations, one with a single CEP X locus on 13 mitoses and 60 nuclei, one with three CEP X locus on six mitoses and 30 nuclei, and the other with two CEP X locus on three mitoses and 10 nuclei) (Figures 1A-1C).

An assessment of comorbidities was requested and returned without any particularities notably with the malformation, autoimmune, and metabolic assessment. The therapeutic decision was to treat the patient with recombinant growth hormone (GHr) and to start low-dose estrogen therapy, after one year of GHr treatment, to optimize pre-pubertal growth under GHr.

## Discussion

Our article describes the case of Turner syndrome, in its mosaic form, with three cell lines (45, X/46, XX/47XXX), discovered in a patient presenting a typical clinical syndrome made of stature-weight growth delay (SWGD), associated with a pubertal delay, and a dysmorphic syndrome.

Turner syndrome is a chromosomal disorder resulting from the partial or total absence of one of the two sex chromosomes (X) normally present in girls. It occurs in one in 2,500-3,000 live births and is the only complete monosomy that is compatible with life [3,6].

Karyotype is the key element to confirm the diagnosis, and the chromosomal deletion may be homogeneous in all cells or mosaics [7,8]. Approximately 50%-60% of patients have complete monosomy of the X chromosome, which is defined as the absence of the second sex chromosome and expressed as 45, X. Additionally, 20%-40% of all cells are mosaics of two or more normal or pathological cell lineages generated from the same zygote (e.g., 45, X/46, XX; 45, X/46, X, i (Xq); 45, X/46, XY) [9].

A small proportion (3%-4%) are mosaics for a triple X cell line (47, XXX) [4,5]. The three-cell lineage mosaicism 45, X/46, XX/47, XXX, that our patient represents, is an atypical and very rare form of Turner syndrome. Only a few cases have been described in the literature and have been reported in studies on Turner syndrome [10].

Clinically, the majority of mosaic patients with a chromosomal constitution 45, X/46, XX/47, XXX, described in the literature, did not have typical clinical signs of Turner syndrome and were not diagnosed at an early age. Therefore, there were some cases with a normal phenotype and many with few symptoms [10].

Among the published cases, with X/XX/XXX mosaicism, only 14% of them had a small size [4]. Patients with mosaicism, for a XXX cell line, with or without the presence of a normal 46, XX component, had a substantially higher likelihood of experiencing spontaneous menarche (70%), compared to women with 45, X, who do not have mosaicism (11%). The spontaneous evolution in the majority of these women is toward secondary amenorrhea or premature menopause [4]. Pregnancy is more likely for women with a cell line 46, XX or 47, XXX, or both, compared to those with monosomy X alone [11].

On the other hand, patients with all the signs of Turner syndrome are very rare in the literature [10]. Our patient is one of these exceptional cases as she presents all the typical signs of TS (SWGD + primary amenorrhea + dysmorphic syndrome) despite three-cell lineage mosaicism (X/XX/XXX).

Establishing phenotype-karyotype correlations in Turner syndrome remains a problem. Almost no prediction of final adult size can be made based on karyotype, especially in patients with chromosomal mosaicism, because the percentage of normal/abnormal cells does not match up across all tissues, and the proportion in one tissue does not always predict the proportion in the others. [4]. And it is difficult to assess the relative contribution of each cell lineage to each organ system.

In our patient, the presence of a typical Turner syndrome phenotype (severe SWGD + primary amenorrhea + dysmorphic syndrome), although she has a karyotype with a mosaic form with three cell lines, may be explained by the variability of the mosaicism between the different tissues with a predominance of abnormal cells in the affected tissues. This is the reason why she has a predominance of the stigmata of the TS compared to other patients with the same mosaicism.

## Conclusions

Based on our case and existing literature reviews, we suggest that girls with mosaic 45, X/ 46, XXX/ 47, and XXX may have a normal phenotype, without SWGD or puberty, as they may be minimally symptomatic or even have a typical SD phenotype. Therefore, further studies, adding cases with a 45, X/46, XX/47, XXX karyotype, will be necessary to determine the phenotype of TS with a triple lineage containing 47, XXX and to better establish clear recommendations for the therapeutic management of these patients as well as for genetic counseling.
